# Magnetic Resonance Imaging Features of Spontaneous Subdural Hematoma Secondary to Necrotizing Encephalitis in a Dog

**DOI:** 10.1111/vru.70094

**Published:** 2025-09-29

**Authors:** Eunjee Kim, Gyuhyun Kim, Kyoungwon Seo, Junghee Yoon, Jihye Choi

**Affiliations:** ^1^ Department of Veterinary Medical Imaging College of Veterinary Medicine and Research Institute For Veterinary Science Seoul National University Seoul South Korea; ^2^ Laboratory of Internal Medicine College of Veterinary Medicine and Research Institute For Veterinary Science Seoul National University Seoul South Korea

**Keywords:** canine, chronic hemorrhage, diagnostic imaging, meningoencephalitis of unknown origin, spontaneous hemorrhage

## Abstract

A 4‐year‐old castrated male Pomeranian dog with a 2‐year history of necrotizing encephalitis (NE) presented for acute neurological deterioration without trauma. Magnetic resonance imaging (MRI) revealed a broad crescent‐shaped lesion with mixed signal intensities on T1‐ and T2‐weighted images, hypointense areas on T2* images, contrast enhancement in the outer membrane, and a mass‐like lesion with fluid–fluid layers. The lesion was diagnosed as a chronic subdural hematoma secondary to spontaneous hemorrhage in a dog with NE. A direct causal relationship remains uncertain; however, NE could have contributed to the increased vulnerability of bridging veins. Serial MRI evaluations revealed progression of the lesion. This is the first report describing the MRI features of chronic subdural hematoma in a dog with NE.

**Abbreviations**
ADC: apparent diffusion coefficient
COVID‐19: Coronavirus Disease 2019
CT: computed tomography
DWI: diffusion‐weighted imaging
FLAIR: fluid‐attenuated inversion recovery
MRI: Magnetic resonance imaging
NE: necrotizing encephalitis
PCV: packed cell volume
T1W: T1‐weighted
T2W: T2‐weighted

## 1 Signalment, History, and Clinical Findings

A 4‐year‐old castrated male Pomeranian presented with an acute worsening of neurological symptoms. The dog was tentatively diagnosed with necrotizing encephalitis (NE) 2 years prior, based on clinical signs, signalment, and magnetic resonance imaging (MRI) findings, and was managed with immunosuppressive therapy (leflunomide, prednisolone, and clopidogrel) and anticonvulsants (phenobarbital and levetiracetam). Over the past 2 years, the dog had experienced seizures approximately once every 1–2 months, with each episode lasting less than 1 min. Seven months before presentation, the dosages of steroids and anticonvulsants were reduced by approximately 10%. One week before the presentation, the dog had another seizure, followed by progressive deterioration, including the inability to stand or eat independently, respiratory distress, and a decreased level of responsiveness. The patient had no history of trauma or falls.

On neurological examination, the dog exhibited depressed mentation, lateral recumbency, and a rightward head turn. Mixed nystagmus, including rotatory, horizontal, and vertical movements, was observed along with intermittent disconjugate eye movements. The menace response was absent bilaterally, and the palpebral reflex was diminished. The pupils were small and lacked anisocoria. The gag reflex was found to be decreased when the mouth was opened; however, no significant abnormalities were found on physical examination. Blood tests revealed regenerative anemia (PCV [packed cell volume]: 24.7% [reference range: 41%–56.1%]), elevated liver enzymes (alkaline phosphatase: 823 U/L [reference range 14–93.2 U/L]; aspartate transaminase: 46 U/L [reference range 13.7–41 U/L]; alanine transaminase: 66 U/L [reference range 19–77.9 U/L]), normal C‐reactive protein levels (<10 mg/L [reference range 0–20 mg/L]), and a low phenobarbital trough level (6.4 µg/mL [reference range 10–40 µg/mL]).

## 2 Imaging, Diagnosis, and Outcome

A brain MRI was performed to identify the cause of acute neurological deterioration using a 1.5‐T scanner (SIGNA Creator 1.5T; GE Healthcare, Chicago, IL, USA) with an eight‐channel phased‐array knee coil. The dog was placed in sternal recumbency, and the imaging protocol included spin‐echo T2‐weighted (T2W), T1‐weighted (T1W), T2W‐fluid‐attenuated inversion recovery (T2‐FLAIR), T2*, diffusion‐weighted imaging (DWI), apparent diffusion coefficient (ADC), and T1W FLAIR sequences and contrast‐enhanced T1W after intravenous administration of a gadolinium‐based contrast agent (0.01 mmol/kg, Dotarem; Guerbet, Villepinte, France).

**FIGURE 1:**
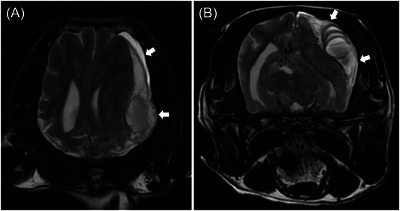
Dorsal (A) and transverse (B) T2‐weighted magnetic resonance images of a subdural hematoma (arrows). A broad, crescent‐shaped lesion is observed in the left cerebral hemisphere. A distinct rightward deviation of the midline and compression of the left cerebral parenchyma and lateral ventricle are observed. There are no signal changes in the surrounding muscle or bone tissues.

**FIGURE 2:**
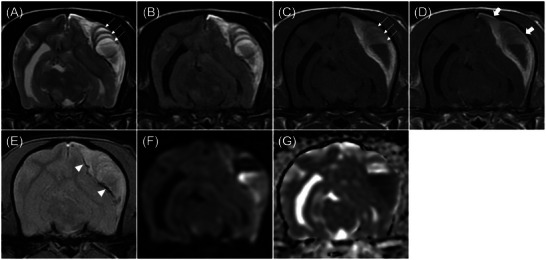
Magnetic resonance transverse images of a subdural hematoma obtained using T2‐weighted imaging (A), FLAIR (B), T1‐weighted imaging (C), T1‐weighted post‐contrast imaging (D), T2* imaging (E), DWI (F), and ADC map (G). The lesion shows mixed intensity on both T2‐weighted (A) and T1‐weighted (C) images. In T2‐ and T1‐weighted images (A and C), hypointense internal septations are located within the lesion (thin arrow). A mass‐like region with fluid levels is observed within the lesion, with two distinct linear layers. The gravity‐dependent layer exhibits low signal intensity on T1‐weighted imaging, hyperintensity on DWI, and hypointensity on the ADC map. The non‐gravity‐dependent layer shows iso‐ to hyperintensity signal on T1‐weighted imaging, hypointensity on DWI, and hyperintensity on the ADC map. Linear signal voids are observed on T2* imaging (arrowhead). Contrast enhancement is observed in the outer membrane adjacent to the cranium (thick arrow). ADC, apparent diffusion coefficient; DWI, diffusion weighted imaging; FLAIR, fluid‐attenuated inversion recovery.

**FIGURE 3:**
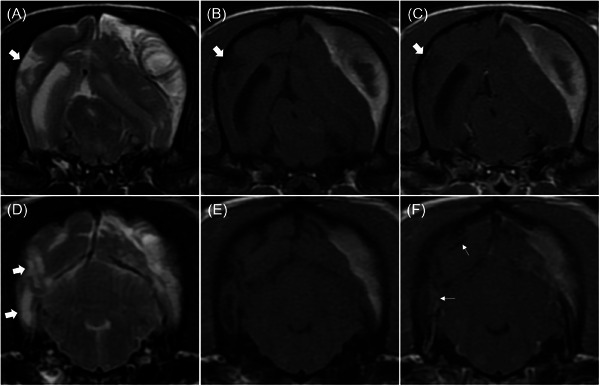
T2‐weighted (A and D), T1‐weighted (B and E), and T1‐weighted post‐contrast (C and F) magnetic resonance transverse images indicating necrotizing encephalitis. Multifocal T2‐weighted hyperintense lesions are observed in the right temporal and occipital lobes. Distinct T2‐weighted hyperintensity and T1‐weighted hypointensity without contrast enhancement indicate necrotic and cystic changes, respectively (thick arrow). T2‐weighted hyperintensity and T1‐weighted isointensity with mild contrast enhancement are observed (thin arrow). And also, additional lesions were located in the left frontal lobe, left temporal, bilateral parietal, piriform lobes, thalamus, pons, and medulla oblongata (not shown).

MRI revealed a broad, crescent‐shaped lesion in the left cerebral hemisphere, causing a rightward midline shift and compression of the left cerebral parenchyma and lateral ventricle (Figure 1). The lesion exhibited mixed intensities on T2W and T1W images, with internal septations and hypointense areas on T2W, T2‐FLAIR, and T2* images. A mass‐like region with fluid levels was observed within the lesion, along with areas of hyperintensity on DWI and hypointensity on the ADC map. Linear signal voids on T2* images indicated the presence of hemorrhage. Contrast enhancement was observed in the outer membrane adjacent to the cranium (Figure 2). These findings were consistent with those of a subdural hematoma. No signal changes were observed in the surrounding muscle or bone tissue. MRI findings consistent with NE were also observed, including multifocal T2W hyperintense lesions in the left frontal and bilateral temporal, parietal, piriform, and occipital lobes, as well as in the thalamus, pons, and medulla oblongata. These lesions predominantly affected the gray matter, with some involvement of the white matter, and showed necrotic and cystic changes (Figure 3).

The dog was tentatively diagnosed with a subdural hematoma secondary to NE. To manage the clinical signs, intracranial pressure control was initiated with an intravenous injection of dexamethasone (0.07 mg/kg, once daily) and mannitol (0.5 g/kg, twice daily), along with anticonvulsant therapy using phenobarbital (4 mg/kg loading dose, every 4 h, total: 16 mg/kg) and levetiracetam (30 mg/kg, thrice daily). These treatments resulted in slight improvements in mentation and pupil size and reduced nystagmus frequency.

**FIGURE 4:**
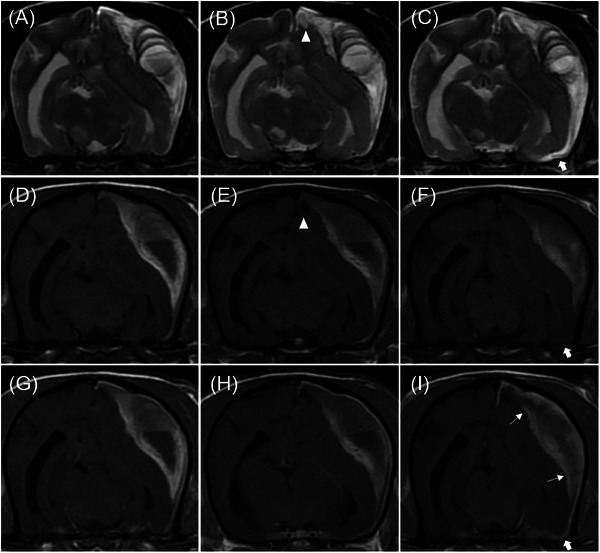
The serial magnetic resonance images were obtained on Day 0 (A, D, and G), Day 6 (B, E, and H), and Day 15 (C, F, and I), using T2‐weighted (A–C), pre‐contrast T1‐weighted (D–F), and post‐contrast T1‐weighted (G–I) sequences. The necrotizing encephalitis lesions in the right temporal lobe, thalamus, and left parietal lobe adjacent to the falx cerebri show increased cystic changes (arrowhead) on Days 6 and 15 compared to Day 0. On Day 15, subdural hematoma lesions are seen to slightly extend into the ventral part of the piriform lobe and the falx cerebri (thick arrow). Contrast enhancement of the inner membrane is also observed (thin arrow).

On Day 6 after the first MRI scan, the dog still showed obtunded mentation, lateral recumbency, a rightward head turn, and an absent bilateral menace response. However, the palpebral reflex was improved, the gag reflex was normalized, the nystagmus frequency was reduced, and the PCV was 23.9%, which was similar to the previous measurement. Repeat MRI on Day 6 revealed that the shape, signal intensity, and size of the subdural hematoma had remained consistent with the previous findings (Figure 4). The NE lesions in the right temporal lobe, thalamus, and left parietal lobe adjacent to the falx cerebri showed increased cystic changes. T2W hyperintense lesions in the pons and medulla oblongata were reduced in size, suggesting alleviation of the associated edema.

On Day 15, the dog showed improvement in alert mentation, reduced rightward head turn, and resolution of nystagmus. Although the dog was unsteady, it could stand independently. MRI on Day 15 revealed persistent midline shift and compression of the left cerebral hemisphere and lateral ventricle. However, the subdural hematoma lesions had extended slightly into the ventral part of the piriform lobe and the falx cerebri. Decreased signal void areas on T2* imaging and increased contrast enhancement along the left cerebellar margin and inner membrane of the hematoma were observed. The other lesions did not change significantly.

Follow‐ups were conducted until Day 130 after the initial onset of symptoms. Until now, the frequency of seizures has remained stable at approximately twice per month, and improvements in the menace response and postural reactions have been maintained.

This case report was conducted with the consent of the owner. This study was approved by the Seoul National University Veterinary Medical Teaching Hospital, and the dogs were managed in accordance with the Guidelines for Animal Experiments of Seoul National University Veterinary Medical Teaching Hospital.

## 3 Discussion

Our case provided details of serial MRI changes during the pathophysiological progression of a chronic subdural hematoma, allowing for direct visualization of the lesion changes and precise monitoring of lesion progression. Serial MRI examinations offered a comprehensive understanding of lesion development, particularly in a dog with NE. To the best of our knowledge, this is the first report describing the MRI features of a spontaneous chronic subdural hematoma in a dog with NE.

In this case, the subdural hematoma was visualized as a broad, crescent‐shaped lesion in the left cerebral hemisphere on MRI, allowing for differentiation from other types of intracranial hematomas. A subdural hematoma is an accumulation of blood between the inner layer of the dura mater and the pia‐arachnoid [1, 2]. Epidural hematomas occur in a well‐defined anatomical space, while the subdural space does not truly exist; instead, subdural hematomas develop within the dural border zone, a structurally fragile region composed of fibroblasts and collagen [3, 4]. Subdural hematomas can occur primarily owing to the rupture of the bridging veins traversing this space, which are particularly vulnerable to injury where they enter the dural venous sinus. Although trauma is a typical cause of subdural hematoma, nontraumatic etiologies can rarely induce spontaneous subdural hematomas. In human medicine, spontaneous subdural hematoma accounts for 0.7%–6.7% of all cases and can result from conditions such as hypertension, vascular malformations, hematological malignancies, infection, hypervitaminosis, and coagulopathy, which weaken the bridging veins or cause a sudden increase in intravascular pressure [5, 6]. During the Coronavirus Disease 2019 (COVID‐19) pandemic, several cases of spontaneous subdural hematoma related to COVID‐19 have been reported [7, 8]. In veterinary medicine, spontaneous subdural hematomas are even rarer, with only two cases reported: one dog with neuronal ceroid lipofuscinosis [9] and one dog with hydrocephalus [10], both of which are associated with brain atrophy. The bridging veins traversing this space are particularly vulnerable to rupture due to shearing forces, especially in conditions associated with cerebral atrophy [6]. In the present case, the history of NE raised concerns about a possible association between NE and the development of a subdural hematoma. NE can result in progressive brain parenchymal atrophy, which may increase the vulnerability of bridging veins. However, a direct causative relationship between NE and the subdural hematoma remains uncertain, and other contributing factors cannot be excluded.

Computed tomography (CT) is the standard diagnostic modality for evaluating subdural hematomas in human medicine, particularly in acute cases; however, MRI is superior to CT for characterizing hematoma composition, internal structures, and associated pathological changes [11, 12, 13]. In acute subdural hematoma, the MRI features are similar to those of intraparenchymal hematoma, including hypointensity on T2W, hypo‐ to isointensity on T1W, marked hypointensity on T2*, and hypointensity on DWI and ADC maps [14, 15].

Over time, chronic subdural hematomas undergo progressive changes due to angiogenesis, fibrinolysis, and inflammation within the dural border zone, leading to blood degradation and membrane formation [16]. These pathological progressions result in distinct MRI features, including mixed signal intensities on T1W and T2W images, internal septations, and mass‐like lesions with outer membrane enhancement, thereby differentiating them from intraparenchymal hematomas [3].

The MRI findings in this case were consistent with those of chronic subdural hematoma, demonstrating a broad, crescent‐shaped lesion with mixed signal intensities on T2W and T1W images; internal septations; hypointense areas on T2W, T2‐FLAIR, and T2* images; and a mass‐like region with outer membrane enhancement. Although spontaneous subdural hematomas are rare, with only a few reported cases in veterinary medicine, our findings were comparable with those reported in a dog with neuronal ceroid lipofuscinosis [9], where MRI revealed a subdural hematoma in both the cerebral and cerebellar regions. The lesion appeared slightly hyperintense on the T1W image with incomplete suppression on the FLAIR sequence, accompanied by a gravity‐dependent fluid line and a susceptibility artifact on the T2* images in the ventral aspect of the fluid [9]. Similar findings have been reported in a study involving 27 human patients, where chronic subdural hematoma showed mixed intensity on T1W and T2W images with internal septations (74%), outer membrane enhancement adjacent to the cranium (100%), and, in some cases, inner membrane enhancement near the brain (33%) [3]. Approximately two‐thirds of cases showed mass‐like enhancement, predominantly visible on FLAIR images and less frequently on T1W images [3]. The majority also exhibited hypointensity on T2*‐gradient echo and/or susceptibility‐weighted imaging sequences, and diffusion restriction was observed in one‐third of cases.

On the initial MRI images of our case, the hematoma grows inward owing to inflammation from the innermost zone of the dura mater, forming a crescent shape. As the lesion progresses, a transition zone between the outer and inner membranes emerges, eventually resulting in a triangular shape. Repeated angiogenesis, fibrinolysis, and persistent inflammation lead to resorption and organization of chronic subdural hematoma [3, 16]. These changes are visualized as internal septations and distinct outer‐ and inner‐membrane enhancements on MRI, reflecting ongoing remodeling and compartmentalization of the hematomas [3, 16, 17]. Membrane proliferation and vascular remodeling play crucial roles in this process, with both the outer and inner membranes consisting of vascular granulation tissue [3, 16]. The outer membrane, which contains more active inflammatory cells than the inner membrane, shows stronger contrast enhancement in the early stages, as observed in our serial MRI scans. This finding indicates an inward expansion of the inflammation during hematoma maturation.

Diffusion restriction along the outer membrane or neomembrane in chronic subdural hematomas may indicate ongoing or recurrent bleeding from a fragile neovascularized membrane. In the distinct two‐layer structure of our case, the non‐gravity‐dependent layers appeared hypointense on DWI and hyperintense on the ADC map, suggesting a low‐cellularity fluid. Conversely, the gravity‐dependent layer was hyperintense on DWI and hypointense on the ADC map, indicating a high‐cellularity fluid containing blood degradation products. The distinct two‐layer structure, separated by a horizontal line, likely reflects the gravity‐dependent sedimentation of hemolysis and inflammatory products.

Over time, serial MRI examinations revealed that the separation between the two layers became less defined, with the gravity‐dependent layer transitioning from isointense to hyperintense on the T2W images and from hypointense to isointense to hyperintense on the T1W images. These changes differ from the typical blood progression of intraparenchymal hematomas, which are confined within brain tissue, whereas the subdural hematomas exist within the dural border zone, where hemosiderin‐laden macrophages can more effectively clear blood degradation products [3, 18]. This difference likely accounts for the unique progression of chronic subdural hematomas compared with aging intraparenchymal hemorrhages. In addition, the lesion showed a gradual increase in contrast enhancement of the inner membrane and a progressive reduction in the distinct fluid–fluid level within the mass‐like lesion over time. These changes could be attributed to the repeated processes of organization and resorption of the hematoma, although the exact mechanisms remain unclear.

As the hematoma matures, the mass‐like appearance can also be visualized as the result of contrast leakage through the inflammatory membranes into the hematoma cavity. Newly formed subdural hematomas tend to enhance faster than older chronic subdural hematomas [3]. Within these mass‐like lesions, fluid–fluid layering may reflect different stages of inflammation and sedimentation, influenced by the varying concentrations of proteins and cellular components. In our case, the lesion showed contrast enhancement limited to the inner and outer membranes, with no enhancing area within the hematoma cavity. However, it demonstrated a well‐defined fluid layer indicative of a hematoma, notably displaying a two‐layer structure.

This study had some limitations. First, a definitive diagnosis of NE was not achieved via histopathology; however, the clinical and imaging findings suggest that NE remains the most probable diagnosis. Second, the use of clopidogrel, an antiplatelet agent, may have influenced the hemorrhage risk. Although intracranial hemorrhage solely due to clopidogrel has rarely been reported [19], its role was unknown in this case. In conclusion, this is the first report of the MRI characteristics of chronic subdural hematoma in a dog with NE and highlights the importance of recognizing spontaneous subdural hematoma as a potential complication in chronic neurological conditions, such as NE. This study also describes the detailed MRI features of chronic subdural hematomas using serial examinations. Serial MRI examinations are crucial for confirming the chronic nature of spontaneous subdural hematomas and tracking their progression over time.

## List of Author Contributions

Category 1

- Conception and Design: E Kim, Choi
- Acquisition of Data: E Kim, G Kim
- Analysis and Interpretation of Data: E Kim, Choi, G Kim, Seo, Yoon

Category 2

- Drafting the Article: E Kim, Choi
- Reviewing Article for Intellectual Content: E Kim, Choi, G Kim, Seo, Yoon

Category 3

- Final Approval of the Completed Article: E Kim, Choi, G Kim, Seo, Yoon

Category 4

- Agreement to be accountable for all aspects of the work in ensuring that questions related to the accuracy or integrity of any part of the work are appropriately investigated and resolved: E Kim, Choi, G Kim, Seo, Yoon

## Disclosure

An abstract has been accepted for the upcoming FAVA conference on October 25–26, 2024. No reporting checklist was used for this study.

## Conflicts of Interest

The authors declare no conflicts of interest.

## Data Availability

Data supporting the results in this article are available from the corresponding author upon a reasonable request.
